# Long‐term evaluation of fitness and demographic effects of a Chinook Salmon supplementation program

**DOI:** 10.1111/eva.12725

**Published:** 2018-11-15

**Authors:** Ilana Janowitz‐Koch, Craig Rabe, Ryan Kinzer, Doug Nelson, Maureen A. Hess, Shawn R. Narum

**Affiliations:** ^1^ Columbia River Inter‐Tribal Fish Commission Hagerman Idaho; ^2^ Department of Fisheries Resources Management Nez Perce Tribe McCall Field Office Idaho

**Keywords:** parentage analysis, reproductive success, salmonids, supplementation

## Abstract

While the goal of supplementation programs is to provide positive, population‐level effects for species of conservation concern, these programs can also present an inherent fitness risk when captive‐born individuals are fully integrated into the natural population. In order to evaluate the long‐term effects of a supplementation program and estimate the demographic and phenotypic factors influencing the fitness of a threatened population of Chinook Salmon (*Oncorhynchus tshawytscha*), we genotyped tissue samples spanning a 19‐year period (1998–2016) to generate pedigrees from adult fish returning to Johnson Creek, Idaho, USA. We expanded upon previous estimates of relative reproductive success (RRS) to include grandparentage analyses and used generalized linear models to determine whether origin (hatchery or natural) or phenotypic traits (timing of arrival to spawning grounds, body length, and age) significantly predicted reproductive success (RS) across multiple years. Our results provide evidence that this supplementation program with 100% natural‐origin broodstock provided a long‐term demographic boost to the population (mean of 4.56 times in the first generation and mean of 2.52 times in the second generation). Overall, when spawning in nature, hatchery‐origin fish demonstrated a trend toward lower RS compared to natural‐origin fish (*p* < 0.05). However, when hatchery‐origin fish successfully spawned with natural‐origin fish, they had similar RS compared to natural by natural crosses (first‐generation mean hatchery by natural cross RRS = 1.11 females, 1.13 males; second‐generation mean hatchery by natural cross RRS = 1.03 females, 1.08 males). While origin, return year, and body length were significant predictors of fitness for both males and females (*p* < 0.05), return day was significant for males but not females (*p* > 0.05). These results indicate that supplementation programs that reduce the potential for genetic adaptation to captivity can be effective at increasing population abundance while limiting long‐term fitness effects on wild populations.

## 1 INTRODUCTION

For species of conservation concern, understanding the fitness effects from captive breeding programs can be critical to their management and long‐term persistence (reviewed in Araki, Berejikian, Ford, & Blouin, 2008; Williams & Hoffman, 2009). Genetic adaptation to captivity, for example, can occur within a small number of generations after introduction to a captive environment (Christie, Marine, Fox, French, & Blouin, 2016; Christie, Marine, French, & Blouin, 2012; Roberge, Normandeau, Einum, Guderley, & Bernatchez, 2008) and give rise to a “domestication phenotype” (reviewed in Jensen, 2006; McDougall, Réale, Sol, & Reader, 2006; Price, 1984). In addition, captive‐born individuals that are released into the wild may exhibit significantly lower fitness than their wild counterparts (Araki, Cooper, & Blouin, 2007; Frankham, 2008; Mathews, Orros, McLaren, Gelling, & Foster, 2005; Miller, Close, & Kapuscinski, 2004).

Owing to habitat degradation and fragmentation, dam construction, overfishing, and climate change, multiple species and populations of Pacific salmon are at risk of extirpation (Gustafson et al., 2007). To reduce the likelihood of extirpation, hatchery supplementation programs have been implemented throughout the Pacific Northwest (USA) to increase population abundance (Naish et al., 2007; Paquet et al., 2011). However, previous research shows that hatchery‐origin fish released into the river systems can affect the fitness of wild stocks (Araki, Cooper, et al., 2007). Although supplementation programs, such as hatchery programs, may cause deleterious fitness effects (Araki, Cooper, & Blouin, 2009; Araki, Cooper, et al., 2007; Christie et al., 2012; Ryman & Laikre, 1991), they can also provide a large demographic boost (DB) to natural populations through an increase in the reproductive success (RS) of the population as a whole (Cuenco, 1994).

For both hatchery‐origin and natural‐origin salmon that spawn in nature, RS can be highly variable among individuals (Ford, Murdoch, Hughes, Seamons, & LaHood, 2016; Hess et al., 2012; Williamson, Murdoch, Pearsons, Ward, & Ford, 2010). Variation in RS can be influenced by numerous behavioral and phenotypic traits that may interact with one another to impact fitness. For example, a strong positive relationship between phenotypic traits such as body length and RS for both males and females has been documented across numerous studies (Berejikian, Doornik, Scheurer, & Bush, 2009; Berntson, Carmichael, Flesher, Ward, & Moran, 2011; Seamons & Quinn, 2010; Seamons, Bentzen, & Quinn, 2004; Serbezov, Bernatchez, Olsen, & Vøllestad, 2010). The timing of arrival on breeding grounds can also affect RS (Berntson et al., 2011; Dickerson, Quinn, & Willson, 2002; Ford et al., 2016). In addition, rearing history (i.e., hatchery‐ vs. natural‐origin) may influence various phenotypic traits and can affect RS (Araki, Ardren, Olsen, Cooper, & Blouin, 2007; Berntson et al., 2011; Ford et al., 2006, 2016; Hess et al., 2012).

Despite increases in salmonid abundance from supplementation programs (Paquet et al., 2011), the use of hatcheries remains controversial. Therefore, there is a growing need for empirically based scientific evaluation of the long‐term effects of hatchery fish on the wild population (Flagg, 2015). Recent evidence suggests that integrating natural‐origin individuals into the hatchery breeding pairs (i.e., broodstock) can boost natural population abundance with minimal negative fitness impacts to the wild population (Hess et al., 2012; Schroder et al., 2008). However, it remains unclear whether these effects are sustained across multiple generations. This study extends results from Hess et al. (2012) to evaluate long‐term fitness effects between hatchery‐origin fish that spawn with natural‐origin fish in nature across multiple generations and examines factors influencing highly variable RS that is observed among individuals. We utilized a 19‐year (1998–2016) span of genetic pedigrees from a Chinook Salmon (*Oncorhynchus tshawytscha*) population that integrates 100% natural‐origin individuals into the hatchery broodstock to specifically (a) expand upon previous work by Hess et al. (2012) and evaluate whether supplementation continued to provide a long‐term DB to the population, (b) test for overall differences in fitness (i.e., RS) between hatchery‐ and natural‐origin fish across two generations, and (c) test the significance of other potential factors (e.g., body length and return timing) affecting fitness in this population.

## 2 METHODS

### 2.1 Study site and sample collection

Over the period of 1998–2016, approximately 14,500 caudal fin samples were collected from Chinook Salmon returning to spawn in Johnson Creek, Idaho, as part of a supplementation program implemented by the Nez Perce Tribe (NPT) in 1998 (see Table S1 for sample sizes). Johnson Creek is a spawning aggregate of the East Fork South Fork Salmon River spring/summer Chinook Salmon population, representing one of 32 evolutionarily significant units of spring/summer Chinook Salmon populations in the Snake River (see Supporting Information Figure S1 for map of the South Fork Salmon River basin). The majority of the tissue samples and biological data used in our analyses were collected from fish trapped at the NPT's Johnson Creek weir, a temporary but highly efficient trap located downriver from where approximately 90% of all redds in Johnson Creek are enumerated (Rabe, Nelson, & Covel, 2016). We also collected tissue from carcasses encountered during spawning ground surveys. Combined, the two approaches were estimated to have sampled a yearly mean of 93% of the entire population.

During sampling, NPT staff recorded data including sex, body length, origin, sampling date, and weir arrival date. Sex was determined by physical morphology. Origin was determined by the presence/absence of coded wire tags, visual implant elastomer tags, or a clipped adipose fin; hatchery‐origin (further referred to as “HOR”) fish in this system have a coded wire tag and/or a visual implant elastomer tag, while natural‐origin (further referred to as “NOR”) fish do not have any markings. Fish reared in this supplementation program assist with recovery of a nearly extirpated population, so no harvest is intended and these HOR fish are not adipose‐clipped. Thus, any adipose‐clipped fish that return to Johnson Creek represent strays from other hatchery programs and are removed at the weir from the spawning population. Arrival time for PIT‐tagged fish was determined through the use of an array mounted on the weir, while arrival time for non‐PIT‐tagged fish was based on weir collection date. For the purposes of this study, data recorded in the field were entered into a shared database and combined with parentage‐informed data. If there were any discrepancies between field‐collected and parentage‐informed data, we deferred to parentage‐informed data. For example, if a fish was listed as a 4‐year‐old from the field‐collected data, but parentage analysis assigned that individual as a 5‐year‐old, we chose the parentage‐informed data to infer age.

As per protocol for this supplementation program, only NOR returns were used as broodstock, which consisted of up to 40 pairs of NOR adults annually. All remaining NOR returns and all Johnson Creek HOR returns were released above the weir for natural spawning. After fertilization of gametes from broodstock pairs, eggs were transferred to the McCall Fish Hatchery, where juveniles were reared for approximately 19 months. Supplementation juveniles were then direct‐released into Johnson Creek. Upon maturation, adults returned to spawn at Johnson Creek at either 3 (male “jacks”), 4, or 5 years old. Female 3‐year‐olds along with 2‐ and 6‐year‐olds returning adults represent a small proportion of returning fish (<1%) and were therefore not included in any analyses due to low sample sizes.

### 2.2 Parentage analysis

**Figure 1:**
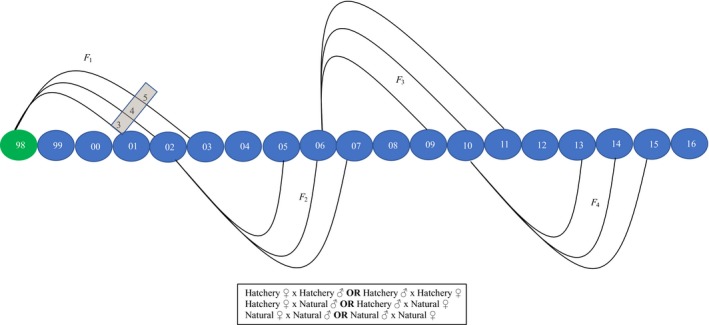
Diagram representing the relationship between return years in this study following four, full generations. Green‐filled circle denotes the start of supplementation in 1998. First‐generation (F_1_) hatchery‐origin fish returned to spawn in nature in 2001, 2002, and 2003 (denoted in blue‐filled circles) as 3‐, 4‐, and 5‐year‐olds, respectively (denoted in the gray box), alongside natural‐origin fish. All possible cross types (H × H, N × N, and H × N) first occurred during 2002–2003 (black box) and could occur between males and females in both directions. Because 2001 was compromised solely of jack male returns, only H × N and N × N crosses were possible that year

The current study extends the parentage results of Hess et al. (2012) that presented genetic‐based pedigrees for adults sampled between 1998 and 2010, with the first generation of returning adults beginning in 2001 (Figure 1). That study provided parentage analyses for HOR and NOR fish that spawned in nature between 2002 and 2005 (Hess et al., 2012). New data presented here represent additional samples collected from 2009 to 2016 that inform parentage results for HOR and NOR fish that spawned in nature from 2006 to 2011 (Figure 1). Overall, for models evaluating factors affecting RS, we utilized all genetic pedigree data spanning 1998–2016 that provided estimates of RS over 10 years (2002–2011).

We extracted genomic DNA from fin tissue using two separate methods over the course of the 19‐year study: a standard Qiagen DNeasy protocol paired with a Qiagen 3000 robotic pipetting system (Qiagen Inc., Valencia, CA) and a Chelex 100 method (Sigma‐Aldrich, St Louis, MO). Genotyping methods transitioned from 13 microsatellite markers to 298 single‐nucleotide polymorphisms (SNPs) throughout the course of the 19‐year study period, but the transition was done in a manner such that no gaps existed in parentage (Table S1). Microsatellite genotyping was completed following methods described previously (Hess et al., 2012), while SNP genotyping was done for a panel of 298 markers with genotyping in thousands by sequencing (GT‐seq; Campbell, Harmon, and Narum (2015); SNP markers described in Hess, Campbell, Matala, Hasselman, & Narum, 2015). In order to transition between marker panels, there were 5 years (2008–2012) of adult returns that were genotyped with both microsatellite and SNP marker panels (Table S1). Specifically, adult returns sampled between 1998 and 2007 were genotyped using only microsatellite markers (see Hess et al., 2012, for details), adult returns sampled between 2008 and 2012 were genotyped with both microsatellites and SNPs, and adult returns from 2013 to 2016 were genotyped using only SNP markers (Table S1).

Single‐nucleotide polymorphism loci with <90% coverage across individuals (i.e., any locus that had ≥10% missing data) were removed prior to running parentage analyses. Since parentage analyses were conducted on a yearly basis, the analyses were split, thereby removing a distinct, data‐specific number of SNPs for each analysis. Therefore, the number of SNPs used for parentage analyses ranged from 276 to 293. In addition, we removed any individual with ≥10% missing genotypes. We then used the package idfgen in R (RCore, 2016) to format data for parentage analysis and remove duplicate samples (https://github.com/mackerman44/idfgen) and the program SNPPIT to assign parentage for adult offspring (Anderson, 2010). The program SNPPIT identifies parent pairs with biallelic markers using a likelihood‐based categorical assignment method and Monte Carlo simulations to assess confidence in assignments. We allowed for a 1% per locus genotyping error rate as recommended by the author (Anderson, 2010), but observed genotyping error based on concordance of quality control tests (i.e., repeated genotyping) was estimated to be <0.1%. Therefore, the estimate used in our analyses was a conservative overestimate of observed error. We did not include sex of the parent in the parentage analysis.

We then removed trio parentage assignments at a false discovery rate (FDR) >0.05 and >2 trio mismatches (Anderson & Garza, 2006; Benjamini & Hochberg, 1995). Additionally, we removed assignments in which the trio having the highest posterior probability of parentage did not involve the two true parents of the offspring (e.g., the candidate parents may be related to the true parents but are not the actual true parents themselves; Anderson, 2010; Hess, Campbell, Docker, et al., 2015). We also removed HOR offspring assigning to nonbroodstock parents, offspring assigning to one broodstock and one nonbroodstock parent, and offspring with spurious assignments to two same‐sex parents. Finally, we determined the overall assignment success for offspring from return year 2013 and 2014, as all parental brood years that produced fish returning in 2013 and 2014 were sampled.

### 2.3 Demographic boost

To determine whether the supplementation program provided a DB to the naturally spawning population, we compared the RS of F_0_ fish taken from the wild and used as hatchery broodstck (RS_BS_) and the RS of F_0_ NOR natural spawners (RS_NS_)

$$F_{0}\text{DB}=\frac{{\text{RS}}_{\text{BS}}}{{\text{RS}}_{\text{NS}}}$$

F_1_ natural spawners that originated from F_0_ broodstock parents were classified as HOR, while F_1_ natural spawners that originated from F_0_ NOR natural spawners were considered NOR. In order to evaluate the DB through the first generation of NOR spawners, it was necessary to account for the F_1_ NORs that returned to Johnson Creek but were removed and used for broodstock. We did this by subtracting the accordant number of F_1_ fish that were used for broodstock and then determining the RS of HOR and NOR fish for the appropriate years

$$F_{1}\text{DB}=\left(F_{0}\text{DB}\right)\left(\frac{{\text{RS}}_{\text{HOR}}}{{\text{RS}}_{\text{NOR}}}\right)$$

This was a more conservative estimate (lower values) of F_1_DB than if we had not accounted for F_1_ fish that were used for broodstock. Overall, this estimate allowed us to measure the RS of grandparents and subsequent second‐generation DB. Males (including jacks) and females were combined for both estimates of F_0_DB and F_1_DB.

### 2.4 Relative reproductive success

To measure lifetime RS, we estimated the number of returning adult offspring for each parent. Females (4‐ and 5‐year‐old adults), males (4‐ and 5‐year‐old adults), and jacks (3‐year‐old males) were analyzed separately. We then compared RS between naturally spawning HOR and NOR fish in 2006–2011 following the methods described in Hess et al. (2012), also including any updates of estimates from previously published results (years 2002–2005). Briefly, we calculated relative reproductive success (RRS) by dividing the average RS of HOR fish by the average RS of NOR fish. RRS estimates were analyzed using two approaches. For the first approach, we included all potential candidate spawners in the population, regardless of whether they were assigned as parents to returning adult offspring. This approach has been used across multiple studies of RRS in salmonids (Araki, Ardren, et al., 2007; Araki, Cooper, et al., 2007; Berntson et al., 2011; Milot, Perrier, Papillon, Dodson, & Bernatchez, 2013; Theriault, Moyer, Jackson, Blouin, & Banks, 2011; Williamson et al., 2010). However, there are numerous reasons that individuals do not produce returning adult offspring, including but not limited to prespawn mortality or staying of offspring (Reviewed in Bowerman, Keefer, & Caudill, 2016; Keefer & Caudill, 2014). Because those individuals do not pass on their alleles to the next generation, we used a secondary approach to estimate the RS of those individuals that did pass their alleles on to the next generation, thereby affecting the long‐term fitness of the natural population. For this secondary approach, we included only those spawners that successfully produced returning adult progeny, removing those individuals that produced zero returning adult offspring (Hess et al., 2012). We found that the difference in genotyping failure rates for HOR versus NOR parents was not significant (ANOVA; *p* = 0.13), and thus, differences in RS were expected to be biological in nature.

Finally, to evaluate fitness effects of HOR fish mating with NOR fish in nature (Araki et al., 2008; Ford et al., 2016; Hess et al., 2012), RS of HOR fish spawning in nature with NOR fish (HOR♀ × NOR♂ or NOR♀ × HOR♂) was compared to NOR × NOR matings. This comparison of cross types allowed us to generate a separate RRS value for the effect of the female parent and the male parent having been reared in the hatchery (Figure 1). We also used assignments to grandparents to compare the RS of HOR × NOR matings to NOR × NOR matings across two full generations for a subset of return years (2002–2006). Similar to our analysis of DB, we subtracted the accordant number of F_1_ fish that were used for broodstock when determining the RS of grandparents. Additionally, we compared HOR × HOR to NOR × NOR matings following similar procedures. Due to low sample sizes, jacks were not included in any estimates involving crosses.

### 2.5 Statistical analyses and phenotypic variation

For each return year and sex, we used ANOVAs to test the null hypothesis that the mean RS was equal for NOR versus HOR fish. We then tested for differences in RS for all four types of crosses H × H, H × N, N × H, and N × N (where female is listed first in each cross type). We also used delta‐method‐based 95% confidence intervals to test for differences in RRS (Bowerman et al., 2016; Ford et al., 2016; Ford, Murdoch, & Howard, 2012; Franz, 2007).

We used generalized linear models (GLMs) to estimate the effects of origin (HOR and NOR) and phenotypes on RS. GLMs were fit to female and male datasets separately and included phenotypic variables of return year, origin, body length, age, and return day. Age was assigned as total age (3, 4, or 5 years old), determined using parentage assignments if available. Return day was the ordinal day a fish was captured at the Johnson Creek weir. Information on origin (hatchery or natural), return year (2002–2011) and day, and body length (fork length measured to the nearest millimeter) was collected at the time of sampling at the weir. Return year, age, and origin were treated as factor variables in GLMs. Return day and body length were transformed to mean absolute deviation estimates to account for variation in return timing and growth among years.

Using a negative binomial distribution model and a log link function, we considered eight candidate models consisting of two to four predictor variables and used AIC model selection, with the best fit model being the one with the lowest AIC score (Akaike, 1992; Burnham & Anderson, 2003). Results of a preliminary exploratory data analysis revealed that quadratic terms did not improve model fit and were therefore not included in the candidate model set. Likewise, interaction terms did not improve model fit and were not included due to limited sample sizes. Additionally, age and body length were highly correlated and not included together in candidate models. Therefore, the full models appear as follows: RS = Return Year + origin + body length + return day or RS = Return Year + origin + age + return day. All GLMs were run in R version 3.3.3 using the *glm.nb* function as part of the MASS package (RCore, 2016; Venables & Ripley, 2002). Graphics were generated using ggplot2, and data manipulation was conducted using dplyr, tidyr, and readr as part of the tidyverse package (Ross, Wickham, & Robinson, 2017; Wickham, 2009).

## 3 RESULTS

### 3.1 Parentage analyses

Parentage analyses for microsatellite data (return years 2002–2007) included previously reported results (return years 2002–2005; Hess et al., 2012), and unpublished analyses for return years 2006–2007, all of which followed the same procedures. For return years 2008–2011, we followed a new protocol for assigning parentage using SNP data. After removing samples based on duplicate genotypes (<0.01% of samples) and coverage, 91% (8,317 of 9,143) remained in the SNP dataset (return years 2008–2016). All parental brood years that produced fish returning in 2013 and 2014 were sampled. Therefore, we used multiple criteria to filter potential false assigned offspring and found a range of assignment success from 76% (2014 offspring) to 77% (2013 offspring). In this study, average LOD of 25.9 and FDR of 0.0002 provided very high confidence in parentage assignments. Within the filtered dataset, the average number of Mendelian mismatches within the trio was 0.30 loci.

### 3.2 Demographic boost and RRS

**Figure 2:**
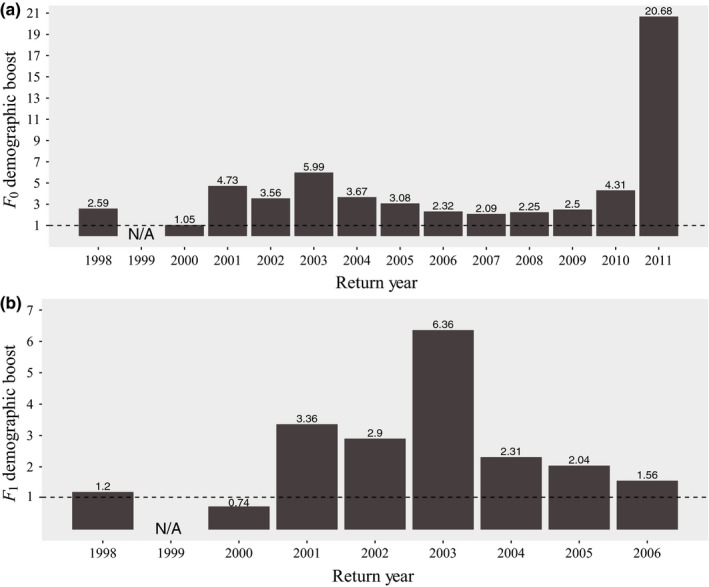
Comparison of the number of offspring produced by fish taken from the wild and used as broodstock and the number of offspring produced by natural‐origin fish spawned in the wild for (a) one generation (F_0_ demographic boost [DB]) and (b) two generations (F_1_ DB). F_0_ DB was calculated by comparing the reproductive success (RS) of broodstock versus naturally spawning fish. F_1_ DB was calculated by comparing the grandoffspring of F_0_ fish after removing F_1_ fish that were used as broodstock. No fish were collected as broodstock in 1999 and are therefore excluded from DB analyses. Fewer return years are presented for the second‐generation results because longer time frames are necessary than first‐generation results (10 vs. 5 years).

**Figure 3:**
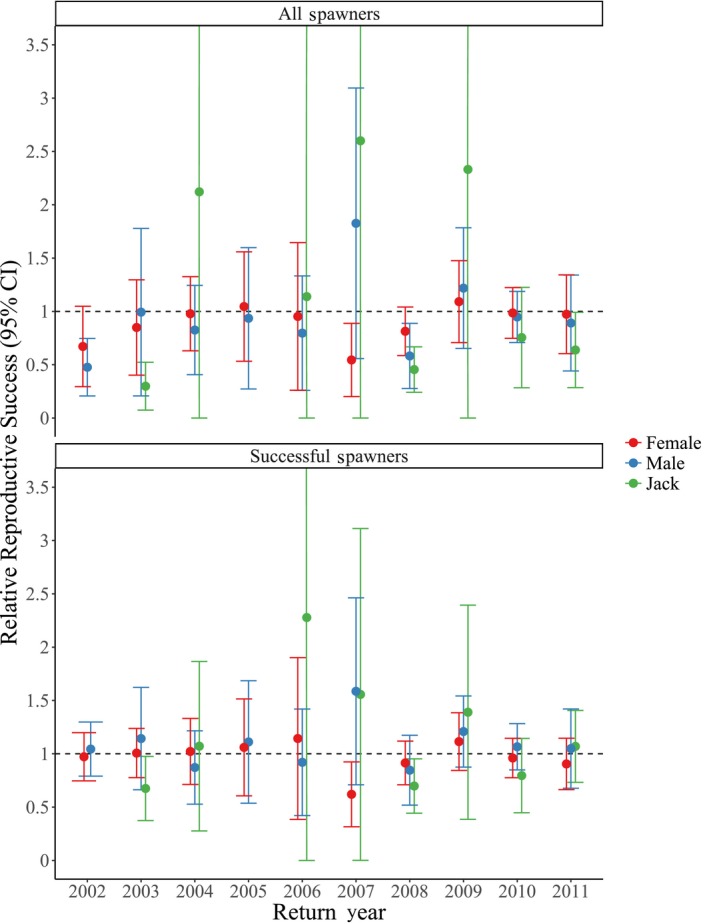
Relative reproductive success (RRS) for return years 2002–2011 for all potential spawners (a) and successful spawners (b). RRS estimates represent the average number of offspring per adult standardized to natural‐origin adult reproductive success (RS) (represented by dashed line). Error bars represent 95% confidence intervals. Due to low sample sizes, jacks from return years 2002 and 2005 were not included in RRS estimates

**Figure 4:**
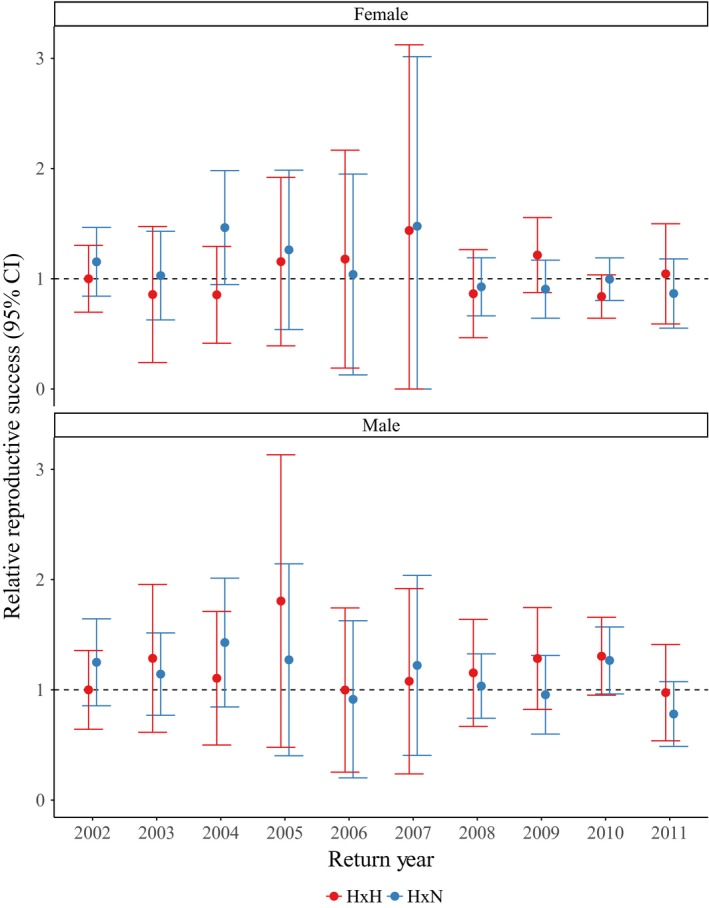
Relative reproductive success (RRS) across one generation for return years 2002–2011 for parental crosses containing at least one hatchery‐origin parent (H × H or H × N) compared to parental crosses involving two natural‐origin parents (N × N). Females (a) and males (b) are presented separately. RRS values are standardized to N × N crosses represented by the dashed line. Error bars represent 95% confidence intervals

**Figure 5:**
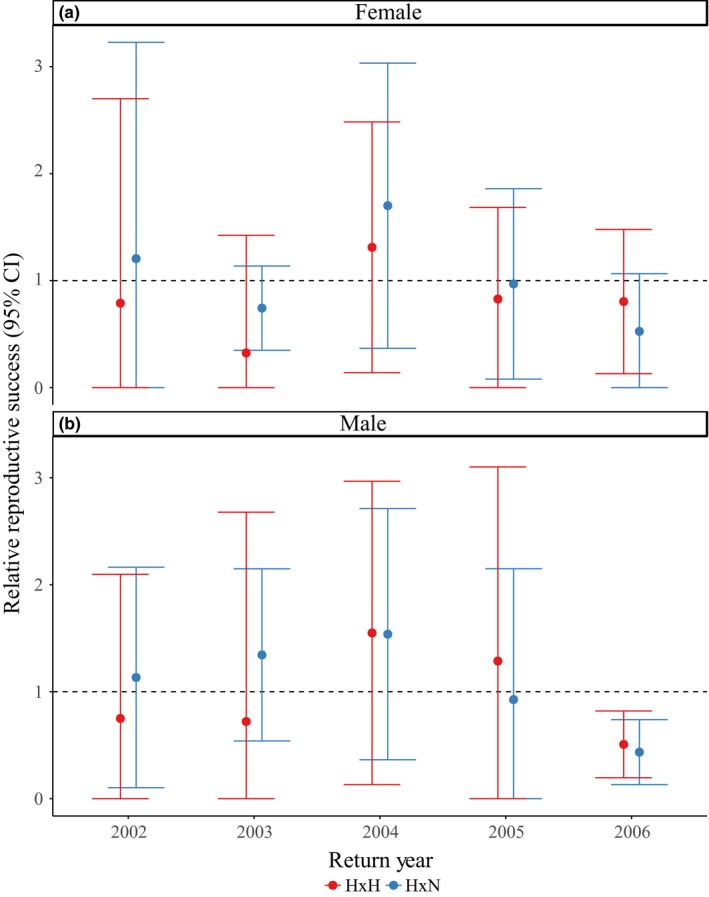
Relative reproductive success (RRS) across two generations for return years 2002–2006 for grandparental crosses containing at least one hatchery‐origin grandparent (H × H or H × N) compared to grandparental crosses involving two natural‐origin grandparents (N × N). Females (a) and males (b) are presented separately. RRS values are standardized to N × N crosses represented by the dashed line. Error bars represent 95% confidence intervals

Across return year 1998–2011, we found a mean (± *SD*) DB of 4.52 (±5.02) from HOR fish for a single generation (Figure 2a) and 2.56 (±1.76) when examining two generations (Figure 2b). After removing outlier values, we found a mean DB of 3.18 (±1.35) after a single generation (return year 2011 removed) and 2.02 (±0.93) when examining two generations (return year 2003 removed). Values of RRS were calculated separately for females (4 and 5 years old), males (4 and 5 years old), and jacks (3 years old; Table S2; Figures 3, 4, 5). When including those naturally spawned individuals that contributed zero returning adult offspring (i.e., all potential candidate spawners), HOR female RRS was significantly lower than 1.0 in one of ten compared return years (return year 2007; Table S2; Figure 3a). HOR male RRS was significantly lower than 1.0 in two of ten compared return years (return year 2002 and 2008) and in two of nine compared return years for jacks (return year 2003 and 2008; Table S2; Figure 3a). When considering all potential candidate females, RRS values ranged from 0.54 to 1.09 (mean = 0.89), 0.48 to 1.83 for all potential candidate males (4 and 5 years old; mean = 0.95), and 0.30 to 2.60 for all potential candidate jacks (mean = 1.30; Table S2; Figure 3a).

When calculating RRS estimates from only those individuals that successfully reproduced (i.e., contributed returning adult offspring), female HOR RRS was significantly lower than 1.0 in one of 10 years (return year 2007; Table S2; Figure 3b), but was not significantly lower for males or jacks for any return years. The RRS values for successful female spawners ranged from 0.62 to 1.14 (mean = 0.97), 0.85 to 1.59 for successful males (4 and 5 years old; mean = 1.09), and 0.68 to 2.28 for successful jacks (mean = 1.19; Table S2; Figure 3b). Interyear variation in RRS remained high, particularly for females (Figure 3).

As stated previously, our evaluations of fitness effects of HOR fish mating with NOR fish were sex‐specific and involved only 4‐ and 5‐year‐olds in RRS estimates (Table S2; Figures 4, 5). Although there were significant differences between H × H versus H × N crosses for females in 2004 and 2009 (Table S2; Figure 4a), there were no significant differences for H × H crosses or H × N crosses compared to N × N crosses for either sex across one generation (Table S2; Figure 4). When measuring the fitness effects of cross types across two generations, we did not find any significant differences for males or females (Table S2; Figure 5). The RRS values across one generation for H × H versus N × N crosses ranged from 0.84 to 1.44 for females (mean = 1.04; Table S2; Figure 4a) and 0.97 to 1.81 for males (mean = 1.20; Table S2; Figure 4b). The RRS values across two generations for H × H versus N × N crosses ranged from 0.32 to 1.31 for females (mean = 0.81; Table S2; Figure 5a) and 0.51 to 1.55 for males (mean = 0.96; Table S2; Figure 5b). The RRS values remained similar for H × N versus N × N crosses across one generation, ranging from 0.87 to 1.48 for females (mean = 1.11; Table S2; Figure 4a) and 0.78 to 1.43 for males (mean = 1.13; Table S2; Figure 4b). Across two generations, the RRS values for H × N versus N × N crosses ranged from 0.53 to 1.70 for females (mean = 1.03; Table S2; Figure 5a) and 0.44 to 1.54 for males (mean = 1.08; Table S2; Figure 5b).

### 3.3 Factors affecting reproductive success

Prior to running GLM analyses, we used exploratory data analyses to determine the distribution of RS (i.e., offspring number) within our dataset. Offspring number demonstrated a negative binomial distribution with the majority of HOR and NOR individuals producing zero offspring, a trend that remained similar for females and males (Supporting Information Figure S2). In addition, return day demonstrated a bimodal distribution that remained consistent across origin type (HOR vs. NOR) and sex (Supporting Information Figure S3).

**Figure 6:**
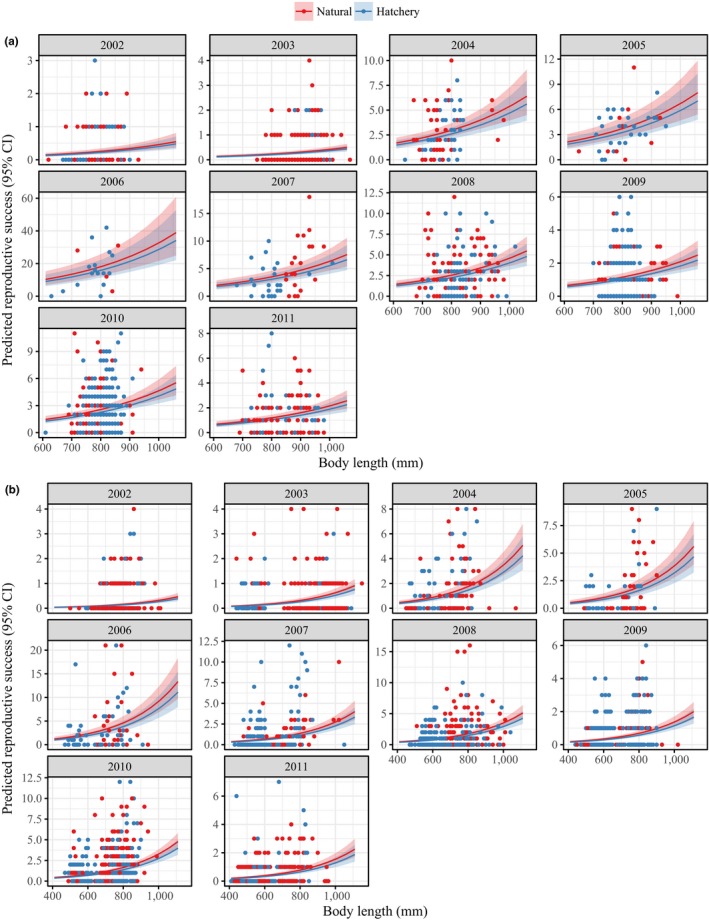
Predicted reproductive success (including regression lines and 95% confidence intervals) holding return day constant and varying body length for (a) females and (b) males. Natural‐origin and hatchery‐origin are depicted as red and blue, respectively

Out of the eight models that we tested, the best fitting model to the data included return year, origin, body length, and day of return for female and male spawners, although three other models had similar AIC scores (Table S3). Overall for females, the estimated coefficients of return years 2004–2011, origin, and body length for predicting RS were significantly different than zero (*p* < 0.05). Return year 2003 and return day were not significant predictors in the model (*p* > 0.05; Table 1). For males, all of the estimated coefficients for predicting RS were significantly different than zero (*p* < 0.01) (Table 1). For both males and females, all significant parameter estimates besides return day demonstrated a positive relationship with RS (Table 1). For example, when holding return day constant, body length demonstrated a positive relationship with RS for both males and females across return years for both HOR and NOR individuals (Table 1; Figure 6).

**Table 1:** Estimated model coefficients for the number of offspring as a function of return year, origin, body length, and return day

	Females		Males	
	Estimate	*SE*	Estimate	*SE*
2003	−0.095	0.198	0.677**	0.177
2004	2.462**	0.182	2.394**	0.174
2005	2.684**	0.209	2.493**	0.202
2006	4.267**	0.227	3.361**	0.185
2007	2.626**	0.201	2.155**	0.163
2008	2.305**	0.165	2.402**	0.148
2009	1.516**	0.168	1.463**	0.155
2010	2.315**	0.156	2.335**	0.140
2011	1.549**	0.177	1.582**	0.163
Origin (NOR)	0.132*	0.063	0.183**	0.065
Body length	0.030**	0.005	0.034**	0.003
Return day	−0.003	0.002	−0.008**	0.002

Note

NOR: natural‐origin.

Males include 3‐, 4‐, and 5‐year‐olds. Coefficients shown are from the best fitting generalized linear model with a negative binomial distribution and a log link function and include estimated standard errors (*SE*) and *p*‐values;

*p < 0.05; **p < 0.01.

## 4 DISCUSSION

Overall, our results demonstrate that the survival advantages conferred to juveniles through the hatchery supplementation program continue to provide a DB to the wild population, providing approximately five times the number of returning adult offspring as naturally spawning parents. When examining the DB through two generations, we see a similar pattern, with HOR fish continuing to provide close to three times the number of adult offspring as NOR fish. As expected, the boost declined between the first and second generation, as HOR fish spawned in the natural environment and their offspring no longer experienced a survival advantage from hatchery rearing. In a small subset of outlier years, most notably 2011, the number of adult offspring from broodstock parents was substantially greater than from naturally spawning parents. These results may reflect an interaction between hatchery effects and other extraneous factors that are unaccounted for in this study, such as abundance. For example, intra‐ and interspecific abundance can affect RS (Achord, Levin, & Zabel, 2003; Essington, Quinn, & Ewert, 2000; Myers, 2001; Quinones, Holyoak, Johnson, & Moyle, 2014) and may interact with factors such as origin to predict RS (Berntson et al., 2011).

Although we demonstrate that RS of HOR and NOR was significantly different for only a small subset of return years (Table S2; Figure 3), we found a general trend of fewer returning adult offspring produced by HOR compared to NOR males (Table S2; Figure 3a). We also show that when accounting for other factors (e.g., return timing, body length), origin predicted RS, with NOR fish demonstrating higher RS than HOR fish (Table 1). These results are consistent with similar findings in other populations (Araki et al., 2008; Christie, Ford, & Blouin, 2014; Ford et al., 2012, 2016). However, negative fitness effects were generally found in populations that incorporated HOR fish into hatchery broodstock. Alternatively, studies utilizing populations that incorporate 100% NOR fish into hatchery broodstock repeatedly demonstrate reduced genetic risks (i.e., domestication selection) associated with supplementation (Fast et al., 2015; Hess et al., 2012; Schroder et al., 2008; Waters et al., 2018, 2015). Therefore, the lack of consistent fitness differences between HOR and NOR fish in this study may reflect the positive effects of a broodstock program that uses only NOR fish. In addition, the majority of other studies assessing fitness differences between HOR and NOR fish have not incorporated other sources of variation that could account for the decrease in fitness of HOR fish (Araki et al., 2008; Christie et al., 2014).

We show that origin, return year, body length, and return day significantly predicted RS for males. For females, return year (with the exclusion of 2003), origin, and body length significantly predicted RS. Overall, body length demonstrated a positive relationship with both male and female RS, a finding that is consistent across salmonid species (Berntson et al., 2011; Ford et al., 2012, 2016; Neff, Garner, Fleming, & Gross, 2015; Seamons, Bentzen, & Quinn, 2007; Williamson et al., 2010). In female salmonids, body size is correlated with greater fecundity and egg size (Dickerson et al., 2002), and in males, large body size is predictive of higher RS, greater access to mates, and increased likelihood of engaging in male–male competition (Berejikian et al., 2009). However, we find that in a subset of return years (e.g., 2007 females), HOR fish exhibit a general trend toward smaller body length compared to NOR fish which may, in part, explain lower HOR RS that same year (Figure 6). Previous research has also found a size difference between HOR and NOR fish that may be reflective of seasonally mediated effects on growth or parental age of maturity through broodstock selection (Hankin, Nicholas, & Downey, 1993; Knudsen et al., 2006; Larsen et al., 2004). The drivers of size differences between HOR and NOR fish were not measured in this study, but interannual differences in environmental conditions could provide a possible explanation and should be addressed in future studies.

Return day demonstrated a significant negative relationship with RS for males, providing evidence that males returning later in the season produce fewer returning offspring. We did not see a significant effect of return day for females in this study. However, the return dates for both sexes show a bimodal distribution with returns either early or late in the season (with a distinct decrease in returns in between the two modes; Supporting Information Figure S3). Although we did not measure selection gradients in the current study, other salmonid studies have demonstrated large, genetically based, heritable variation in return timing that is under distinct selective patterns such as stabilizing or disruptive selection (Anderson, Faulds, Atlas, Pess, & Quinn, 2010; Ford, Hard, Boelts, LaHood, & Miller, 2008; Quinn, McGinnity, Reed, & Bradford, 2016; Quinn, Unwin, & Kinnison, 2000; Seamons et al., 2007). Moreover, other studies have provided support that return date predicts RS, with distinct optimum return dates (Berntson et al., 2011; Kodama, Hard, & Naish, 2012). Therefore, it is possible that return timing in this population is under stabilizing or disruptive selection (Narum, Genova, Micheletti, & Maass, 2018).

Finally, we demonstrate that return year significantly predicted RS, a finding that has been shown in other studies (Berntson et al., 2011; Ford et al., 2016). This finding is also reflected in the large variation in yearly RRS and RS estimates found both in our study and in previous studies (Garant, Dodson, & Bernatchez, 2001; Hess et al., 2012; Jones & Hutchings, 2002; McLean, Seamons, Dauer, Bentzen, & Quinn, 2007; Whiteley et al., 2015). Although unaccounted for in this study, the interaction between annual environmental conditions and phenotypic factors may help to uncover the sources of unexplained variation in RS across years. Other studies in Pacific salmonids have demonstrated an effect of river temperatures on survival and fitness (Hinch et al., 2012). Further, fluctuating ocean conditions, such as upwelling of cold water, can impact fitness in salmonids (Bi, Peterson, Lamb, & Casillas, 2011; Emmett, Krutzikowsky, & Bentley, 2006; Fisher & Pearcy, 1988; Mantua, Hare, Zhang, Wallace, & Francis, 1997; Peterson & Schwing, 2003). Therefore, future studies should be aimed at addressing the specific environmental factors that are associated with variation in RS estimates.

While we show a reduction in fitness of HOR compared to NOR fish for a subset of return years, crosses involving either one or two successful HOR parents demonstrated RS that was not significantly lower than those crosses involving two successful NOR parents (Table S2; Figure 4). These results provide support that fitness generally did not decrease for NOR fish when mating with HOR fish. When we extend RRS estimates of cross types to two full generations, we continue to see a nonsignificant difference between crosses involving either one or two HOR grandparents compared to those involving two NOR grandparents (Table S2; Figure 5).

It is important to note, however, that crosses involving jacks were removed due to low sample sizes. Low sample sizes can result in both imprecise RRS estimates and statistical power that is too low to detect a fitness difference, particularly between crosses (Christie et al., 2014). Although sample sizes in this study varied across years, Hess et al. (2012) provided evidence that an increase in sample size (by combining sexes across years) did not change results of their study nor did removal of years with low sample sizes. In addition, we provide multiple replicates (i.e., return years) with sample sizes that are equal to or greater than previous RRS studies (Araki, Ardren, et al., 2007; Araki, Cooper, et al., 2007; Berntson et al., 2011; Milot et al., 2013; Theriault et al., 2011). Future studies will aim to include single‐parent assignments to increase overall offspring assignment success and subsequent sample sizes for RRS estimates.

While we demonstrate lower RS of HOR fish overall, we do not find a short‐ or long‐term fitness reduction when HOR fish interbreed with the wild population. Other factors in addition to origin, such as return timing and body length, also predict RS in this population. By broadening our understanding of the specific factors affecting fitness of threatened species, we can more efficiently tailor conservation management strategies to focus on maintaining genetic diversity and increasing supplementation as a means of reducing the likelihood of extirpation.

## CONFLICT OF INTEREST

None declared.

## Data Availability

GT‐seq primer sequences, the R script used to analyze data, and both phenotype and genotype data of all fish used in the analysis are available on the Dryad Digital Repository https://doi.org/10.5061/dryad.q6c9891.
