# Supplementary material for: Long‐term evaluation of fitness and demographic effects of a Chinook Salmon supplementation program
Source: Evol Appl. 2018 Nov 15;12(3):456–69. doi: 10.1111/eva.12725 (PMC6383734; doi:10.1111/eva.12725)
Supplement: Supplementary file 1 [file EVA-12-456-s001.docx]

### Supplementary Files

### Table S1. Original escapement estimates and number of hatchery-origin (HOR) and natural-origin (NOR) fish that were used for parentage analyses in this study, separated by return year. MSAT represents years genotyped with microsatellite markers, while SNP indicates years genotyped with SNPs (single nucleotide polymorphisms). Note: column representing the number of fish used for parentage analysis also reflects those individuals with their origin designation changed as a result of parentage analyses (approximately 3% of samples).

| **Return year** |  | **Origin** |  |  | **Escapement** | **Number included in analyses** |  | **Marker type** |
| --- | --- | --- | --- | --- | --- | --- | --- | --- |
| 1998 |  | HOR |  |  | N/A | N/A |  | MSAT |
|  |  | NOR |  |  | 182 | 130 |  | MSAT |
| 1999 |  | HOR |  |  | N/A | N/A |  | MSAT |
|  |  | NOR |  |  | 56 | 15 |  | MSAT |
| 2000 |  | HOR |  |  | N/A | N/A |  | MSAT |
|  |  | NOR |  |  | 186 | 146 |  | MSAT |
| 2001 |  | HOR |  |  | 240 | 239 |  | MSAT |
|  |  | NOR |  |  | 1294 | 1220 |  | MSAT |
| 2002 |  | HOR |  |  | 447 | 429 |  | MSAT |
|  |  | NOR |  |  | 803 | 746 |  | MSAT |
| 2003 |  | HOR |  |  | 206 | 189 |  | MSAT |
|  |  | NOR |  |  | 645 | 584 |  | MSAT |
| 2004 |  | HOR |  |  | 139 | 117 |  | MSAT |
|  |  | NOR |  |  | 252 | 204 |  | MSAT |
| 2005 |  | HOR |  |  | 69 | 64 |  | MSAT |
|  |  | NOR |  |  | 147 | 129 |  | MSAT |
| 2006 |  | HOR |  |  | 87 | 72 |  | MSAT |
|  |  | NOR |  |  | 124 | 102 |  | MSAT |
| 2007 |  | HOR |  |  | 238 | 218 |  | MSAT |
|  |  | NOR |  |  | 189 | 160 |  | MSAT |
| 2008 |  | HOR |  |  | 373 | 356 |  | MSAT & SNP |
|  |  | NOR |  |  | 338 | 318 |  | MSAT & SNP |
| 2009 |  | HOR |  |  | 578 | 520 |  | MSAT & SNP |
|  |  | NOR |  |  | 319 | 280 |  | MSAT & SNP |
| 2010 |  | HOR |  |  | 535 | 492 |  | MSAT & SNP |
|  |  | NOR |  |  | 605 | 549 |  | MSAT & SNP |
| 2011 |  | HOR |  |  | 379 | 295 |  | MSAT & SNP |
|  |  | NOR |  |  | 581 | 473 |  | MSAT & SNP |
| 2012 |  | HOR |  |  | 237 | 233 |  | MSAT & SNP |
|  |  | NOR |  |  | 669 | 520 |  | MSAT & SNP |
| 2013 |  | HOR |  |  | 350 | 335 |  | SNP |
|  |  | NOR |  |  | 780 | 679 |  | SNP |
| 2014 |  | HOR |  |  | 570 | 570 |  | SNP |
|  |  | NOR |  |  | 1102 | 1024 |  | SNP |
| 2015 |  | HOR |  |  | 479 | 469 |  | SNP |
|  |  | NOR |  |  | 529 | 488 |  | SNP |
| 2016 |  | HOR |  |  | 187 | 186 |  | SNP |
|  |  | NOR |  |  | 532 | 530 |  | SNP |
| **Total** |  |  |  |  | 14447 | 13081 |  |  |

**Table S2.** Relative reproductive success (RRS) for all potential spawners, successful spawners, and crosses (one and two generations). Due to low sample sizes, jacks from return year 2002 and 2005 were not included in single-spawner RRS estimates and no jacks were included in cross RRS estimates. *P*-values were calculated from ANOVAs; *p*<0.05 represented in bold.

|  | **Return Year** |  | **RRS** | ***P*-value** |  |  |  |  |  |
| --- | --- | --- | --- | --- | --- | --- | --- | --- | --- |
| **All Potential Spawners** |  |  |  |  |  |  |  |  |  |
| Females | 2002 |  | 0.67 | 0.19 |  |  |  |  |  |
|  | 2003 |  | 0.85 | 0.53 |  |  |  |  |  |
|  | 2004 |  | 0.98 | 0.90 |  |  |  |  |  |
|  | 2005 |  | 1.05 | 0.86 |  |  |  |  |  |
|  | 2006 |  | 0.95 | 0.90 |  |  |  |  |  |
|  | 2007 |  | 0.54 | **0.04** |  |  |  |  |  |
|  | 2008 |  | 0.81 | 0.14 |  |  |  |  |  |
|  | 2009 |  | 1.09 | 0.62 |  |  |  |  |  |
|  | 2010 |  | 0.99 | 0.91 |  |  |  |  |  |
|  | 2011 |  | 0.97 | 0.89 |  |  |  |  |  |
| *Average RRS* |  |  | *0.89* |  |  |  |  |  |  |
| Males | 2002 |  | 0.48 | **<0.01** |  |  |  |  |  |
|  | 2003 |  | 0.99 | 0.99 |  |  |  |  |  |
|  | 2004 |  | 0.83 | 0.45 |  |  |  |  |  |
|  | 2005 |  | 0.94 | 0.85 |  |  |  |  |  |
|  | 2006 |  | 0.80 | 0.50 |  |  |  |  |  |
|  | 2007 |  | 1.83 | 0.06 |  |  |  |  |  |
|  | 2008 |  | 0.58 | **0.02** |  |  |  |  |  |
|  | 2009 |  | 1.22 | 0.39 |  |  |  |  |  |
|  | 2010 |  | 0.95 | 0.68 |  |  |  |  |  |
|  | 2011 |  | 0.89 | 0.65 |  |  |  |  |  |
| *Average RRS* |  |  | *0.95* |  |  |  |  |  |  |
| Jacks | 2003 |  | 0.30 | **<0.01** |  |  |  |  |  |
|  | 2004 |  | 2.12 | 0.23 |  |  |  |  |  |
|  | 2006 |  | 1.14 | 0.94 |  |  |  |  |  |
|  | 2007 |  | 2.60 | 0.15 |  |  |  |  |  |
|  | 2008 |  | 0.45 | **0.01** |  |  |  |  |  |
|  | 2009 |  | 2.33 | 0.16 |  |  |  |  |  |
|  | 2010 |  | 0.75 | 0.42 |  |  |  |  |  |
|  | 2011 |  | 0.64 | 0.10 |  |  |  |  |  |
| *Average RRS* |  |  | *1.30* |  |  |  |  |  |  |
| **Successful Spawners** |  |  |  |  |  |  |  |  |  |
| Females | 2002 |  | 0.97 | 0.81 |  |  |  |  |  |
|  | 2003 |  | 1.01 | 0.95 |  |  |  |  |  |
|  | 2004 |  | 1.02 | 0.89 |  |  |  |  |  |
|  | 2005 |  | 1.06 | 0.79 |  |  |  |  |  |
|  | 2006 |  | 1.14 | 0.69 |  |  |  |  |  |
|  | 2007 |  | 0.62 | **0.04** |  |  |  |  |  |
|  | 2008 |  | 0.91 | 0.43 |  |  |  |  |  |
|  | 2009 |  | 1.11 | 0.37 |  |  |  |  |  |
|  | 2010 |  | 0.96 | 0.69 |  |  |  |  |  |
|  | 2011 |  | 0.90 | 0.46 |  |  |  |  |  |
| *Average RRS* |  |  | *0.97* |  |  |  |  |  |  |
| Males | 2002 |  | 1.04 | 0.73 |  |  |  |  |  |
|  | 2003 |  | 1.14 | 0.55 |  |  |  |  |  |
|  | 2004 |  | 0.87 | 0.49 |  |  |  |  |  |
|  | 2005 |  | 1.11 | 0.70 |  |  |  |  |  |
|  | 2006 |  | 0.92 | 0.76 |  |  |  |  |  |
|  | 2007 |  | 1.59 | 0.08 |  |  |  |  |  |
|  | 2008 |  | 0.85 | 0.38 |  |  |  |  |  |
|  | 2009 |  | 1.21 | 0.17 |  |  |  |  |  |
|  | 2010 |  | 1.07 | 0.54 |  |  |  |  |  |
|  | 2011 |  | 1.05 | 0.80 |  |  |  |  |  |
| *Average RRS* |  |  | *1.09* |  |  |  |  |  |  |
| Jacks | 2003 |  | 0.68 | 0.10 |  |  |  |  |  |
|  | 2004 |  | 1.07 | 0.85 |  |  |  |  |  |
|  | 2006 |  | 2.28 | 0.57 |  |  |  |  |  |
|  | 2007 |  | 1.56 | 0.30 |  |  |  |  |  |
|  | 2008 |  | 0.70 | 0.09 |  |  |  |  |  |
|  | 2009 |  | 1.39 | 0.30 |  |  |  |  |  |
|  | 2010 |  | 0.80 | 0.34 |  |  |  |  |  |
|  | 2011 |  | 1.07 | 0.68 |  |  |  |  |  |
| *Average RRS* |  |  | *1.19* |  |  |  |  |  |  |
| **Crosses** |  | ***One Generation*** | **HxH vs. NxN** | **HxN vs. NxN** | ***P*-value** | ***Two Generations*** | **HxH vs. NxN** | **HxN vs. NxN** | ***P*-value** |
| Females | 2002 |  | 1.00 | 1.15 | 0.37 |  | 0.79 | 1.21 | 0.89 |
|  | 2003 |  | 0.86 | 1.03 | 0.88 |  | 0.32 | 0.74 | 0.34 |
|  | 2004 |  | 0.85 | 1.46 | **0.01** |  | 1.31 | 1.70 | 0.31 |
|  | 2005 |  | 1.16 | 1.26 | 0.69 |  | 0.83 | 0.97 | 0.90 |
|  | 2006 |  | 1.18 | 1.04 | 0.89 |  | 0.80 | 0.53 | 0.44 |
|  | 2007 |  | 1.44 | 1.48 | 0.70 |  |  |  |  |
|  | 2008 |  | 0.86 | 0.93 | 0.78 |  |  |  |  |
|  | 2009 |  | 1.21 | 0.91 | **0.01** |  |  |  |  |
|  | 2010 |  | 0.84 | 1.00 | 0.15 |  |  |  |  |
|  | 2011 |  | 1.04 | 0.87 | 0.65 |  |  |  |  |
| *Average RRS* |  |  | *1.04* | *1.11* |  |  | *0.81* | *1.03* |  |
| Males | 2002 |  | 1.00 | 1.25 | 0.17 |  | 0.75 | 1.14 | 0.85 |
|  | 2003 |  | 1.29 | 1.14 | 0.57 |  | 0.72 | 1.34 | 0.60 |
|  | 2004 |  | 1.11 | 1.43 | 0.17 |  | 1.55 | 1.54 | 0.45 |
|  | 2005 |  | 1.81 | 1.27 | 0.24 |  | 1.29 | 0.93 | 0.85 |
|  | 2006 |  | 1.00 | 0.91 | 0.95 |  | 0.51 | 0.44 | 0.08 |
|  | 2007 |  | 1.08 | 1.22 | 0.79 |  |  |  |  |
|  | 2008 |  | 1.15 | 1.03 | 0.81 |  |  |  |  |
|  | 2009 |  | 1.28 | 0.96 | 0.06 |  |  |  |  |
|  | 2010 |  | 1.31 | 1.27 | 0.08 |  |  |  |  |
|  | 2011 |  | 0.97 | 0.78 | 0.39 |  |  |  |  |
| *Average RRS* |  |  | *1.20* | *1.13* |  |  | *0.96* | *1.08* |  |
|  |  |  |  |  |  |  |  |  |  |

**Table S3.** Models of factors predicting reproductive success (RS) for males (including jacks) and females. Akaike’s information criteria (AIC) and Delta AIC (Δ*_i_*) scores are presented in ascending order with lowest scores first. Best-supported models have smaller AIC scores and larger Akaike weights (w*_i_*). All models were fit using a generalized linear model with a negative binomial distribution and a log link function.

| **Dataset** | **Model structure** | **AIC score** | **Δ*_i_*** | **w*_i_*** |
| --- | --- | --- | --- | --- |
| Females | RS = Return Year + origin + $\text{body length}$ + $\text{return }$day | 4970.08 | 0.000 | 0.486 |
|  | RS = Return Year + origin + $\text{body length}$ | 4971.06 | 0.974 | 0.299 |
|  | RS = Return Year + $\text{body length}$ + $\text{return }$day | 4972.47 | 2.389 | 0.147 |
|  | RS = Return Year + $\text{body length}$ | 4974.01 | 3.932 | 0.068 |
|  | RS = Return Year + age | 4988.41 | 18.324 | 0.000 |
|  | RS = Return Year + age + $\text{return }$day | 4988.88 | 18.797 | 0.000 |
|  | RS = Return Year + origin + age | 4989.28 | 19.197 | 0.000 |
|  | RS = Return Year + origin + age + $\text{return }$day | 4989.97 | 19.885 | 0.000 |
| Males | RS = Return Year + origin + body length + $\text{return }$day | 6922.29 | 0.000 | 0.951 |
|  | RS = Return Year + $\text{body length}$ + $\text{return }$day | 6928.23 | 5.941 | 0.049 |
|  | RS = Return Year + origin + $\text{body length}$ | 6941.60 | 19.310 | 0.000 |
|  | RS = Return Year + $\text{body length}$ | 6947.92 | 25.632 | 0.000 |
|  | RS = Return Year + origin + age + $\text{return }$day | 6962.99 | 40.700 | 0.000 |
|  | RS = Return Year + age + $\text{return }$day | 6964.81 | 42.519 | 0.000 |
|  | RS = Return Year + origin + age | 6970.31 | 48.026 | 0.000 |
|  | RS = Return Year + age | 6971.85 | 49.558 | 0.000 |


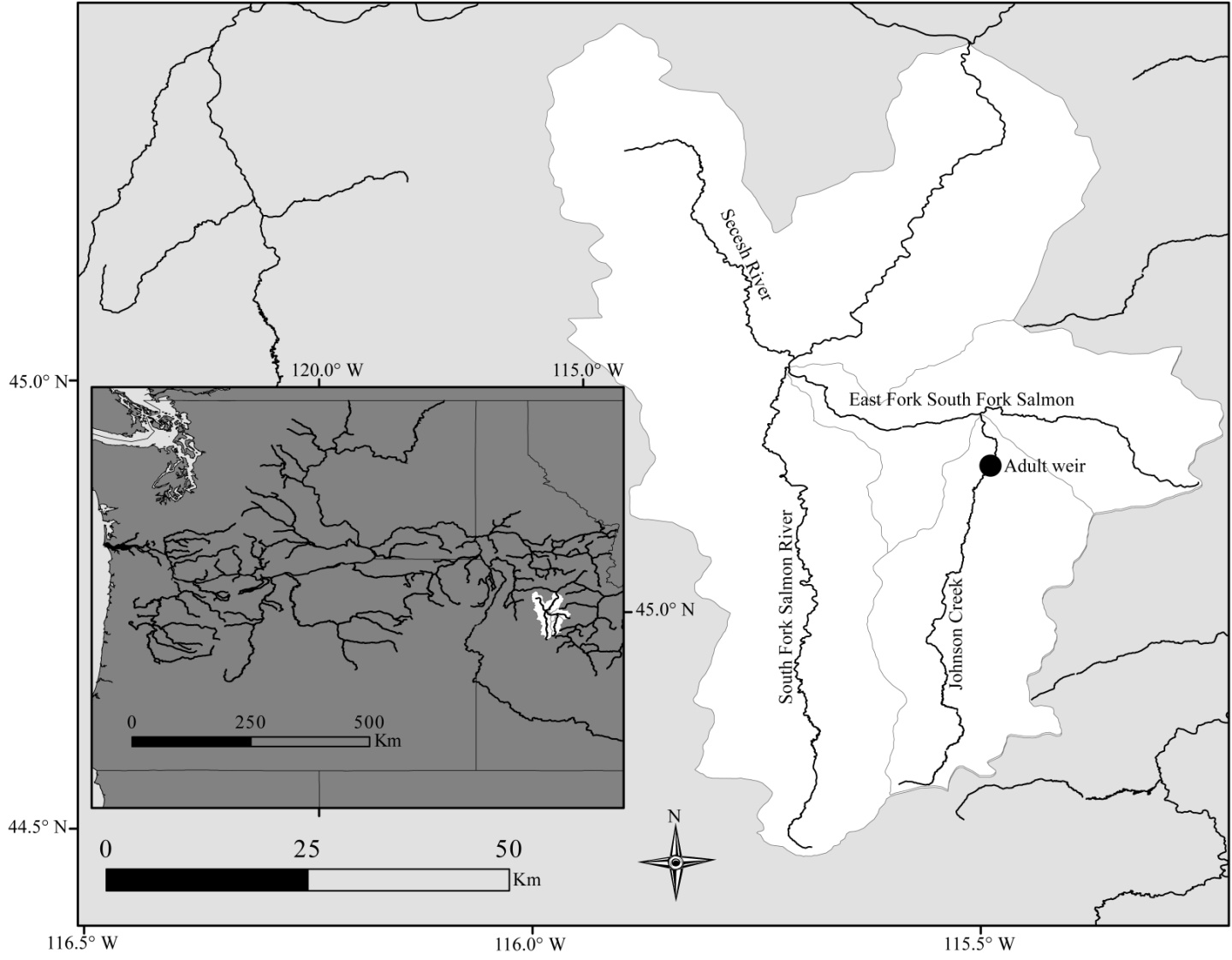
**Figure S1.** Map of the study area and location of Johnson Creek weir. Inset map shows the entire South Fork Salmon River basin (highlighted in white). Reprinted from Hess et al. (2012).

**Figure S2.** Density plots of the number of offspring produced by hatchery- vs. natural-origin fish across return years 2002 through 2011 for females and males, including jacks. Natural- and hatchery-origin are depicted as red and blue, respectively.

**Figure S3.** Density plots of natural- vs. hatchery-origin fish on each return day across years 2002 through 2011 for females and males, including jacks. Return day is presented as ordinal day. Natural- and hatchery-origin are depicted as red and blue, respectively.
